# Stem-cell therapy *via* gastroscopy improves the outcome of esophageal anastomotic leakage

**DOI:** 10.3389/fonc.2022.1077024

**Published:** 2022-12-20

**Authors:** Yannan Hu, Heng Chu, Xiang Xue, Yan Yan, Wenbang Chen, Xilong Lang, Hao Zhang

**Affiliations:** ^1^ Department of Thoracic Surgery, The First Affiliated Hospital, Bengbu Medical College, Bengbu, Anhui, China; ^2^ Department of Thoracic Surgery, Qingdao Municipal Hospital, Qingdao, Shandong, China; ^3^ Department of Cardiothoracic Surgery, The Second Affiliated Hospital, Soochow University, Suzhou, China; ^4^ Department of Cardiothoracic Surgery, No.903 Hospital of Chinese People’s Liberation Army, Hangzhou, Zhejiang, China; ^5^ Department of Cardiovascular Surgery, Changhai Hospital, Naval Medical University, Shanghai, China

**Keywords:** esophageal anastomotic leakage, mesenchymal stromal cells, fibrin scaffold, autograft, gastroscopy

## Abstract

**Background:**

Esophageal anastomotic leakage (EAL) is a severe complication usually occurring after esophagectomy. Although there are various therapeutic methods for EAL treatment, they have not achieved satisfactory results. A previous study showed that the combination of mesenchymal stem cells (MSCs) and fibrin scaffold (FS) can treat EAL. This study aimed to evaluate the efficacy of the injection of MSCs and FS through a new engraftment gastroscope for EAL treatment.

**Methods:**

Twelve adult pigs were randomly divided into the MSCs group (n = 6) and control group (n = 6). A stomach tube was then inserted through the leakage to construct the EAL model, which was removed after one week. The combination of MSCs and FS was autografted at the EAL site for pigs in the MSCs group using the tailor-made gastroscope while only FS was autografted for the pigs in the control group. Local status of EAL was evaluated using gastroscopy. Histological analyses and western blot (WB) were used to assess the gross specimens of esophagi around EALs.

**Results:**

Gastroscopy showed a higher closure rate and a lower infection rate in the MSCs group than in the control group. However, the mortality was not significantly different between the two groups. HE staining showed a severe inflammatory response with dispersive infiltration of inflammatory cells and unhealed leakage in the control group. However, the infiltration of inflammatory cells was not altered in the MSCs group, and the leakage was completely healed. WB analyses showed that Myogenin and α-SMA expressions were significantly higher in the MSCs group than in the control group.

**Conclusion:**

A porcine model of EAL was successfully developed by accessing the transplantation site through the esophagus. Further data revealed that the implantation of MSCs in FS *via* the novel engraftment gastroscope can promote the repair and occlusion of EAL. Therefore, the proposed method is a promising strategy for EAL treatment.

## Introduction

Esophageal anastomotic leakage (EAL) is a severe postoperative complication occurring after esophagectomy. The presence of a tracheoesophageal, mediastinal or esophageal fistula worsens EAL. Food can enter the trachea, chest, mediastinum, and other adjacent parts or organs through the fistula, resulting in severe infection or other lethal complications (1–4). Untreated EAL often leads to death (5–7). The standard treatments for EAL include surgical repair, cervical esophagus exclusion, and esophagectomy. However, the mortality rate for EAL patients is still about 30% (8–12).

Recent advances in endoscopic therapies for EAL include vascular clamps and self-expandable stents. Vascular clamps cause occlusion of the fistula, while self-expandable stents can cover the fistula to avoid aggravating the condition. However, endoscopic treatments are associated with serious complications such as pain, bleeding, migration, and restenosis of a stent (13). Therefore, an effective and safe method for curing EAL is needed.

Mesenchymal stromal cells (MSCs) possess low immunogenicity and induce a good immunosuppressive effect after allograft transplantation. Additionally, the rejection of autologous stem cell transplantation is much milder than that of allogeneic stem cell transplantation (14). Moreover, fibrin is a good carrier of MSCs during transplantation. Xue et al. (2019) demonstrated higher rates of EAL closure in a rabbit model after the implantation of MSCs delivered through the original incision *via* a fibrin scaffold (15).

However, we encountered several problems when trying to reproduce the procedure in pigs *via* MSCs injection through the original neck incisions. First, severe adhesion to tissues and deeper location of EALs in pigs made dissection very complex, resulting in more bleeding. Second, significantly higher titers of inflammatory cells in blood were detected after one week during a routine examination. Third, gastroscopy showed incomplete occlusion of EALs with serious infection around the leakage site after three weeks of MSCs transplantation.

**Figure 1:**
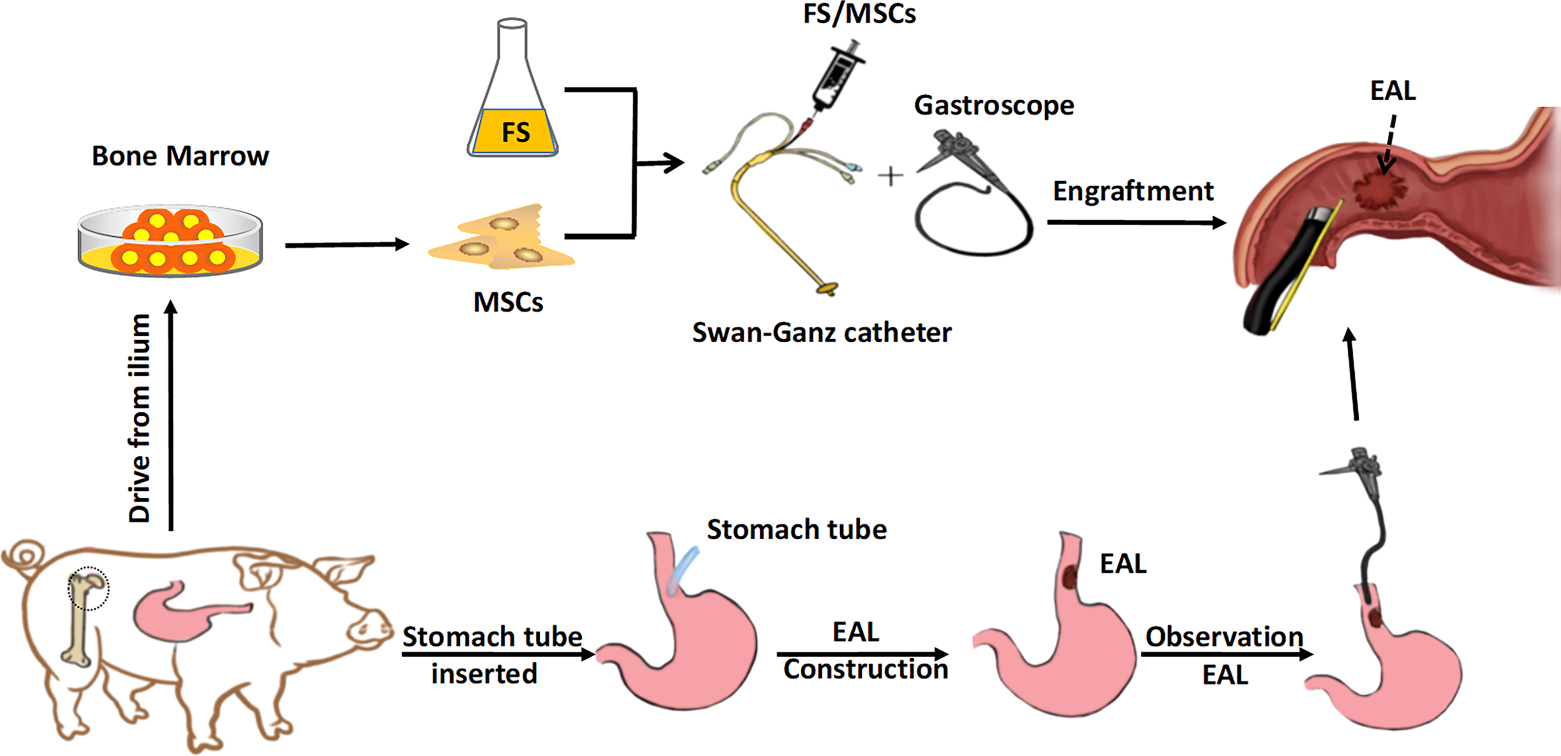
Schematic of the experiment. The MSCs derived from the bone marrow were engrafted in FS *via* a gastroscope for EAL treatment. EAL, esophageal anastomotic leakage; MSCs, mesenchymal stromal cells; FS, fibrin scaffold.

As a result, we combined the gastroscope with a Swan-Ganz catheter to produce a novel equipment for engraftment (**Figure 1**). This study aimed to explore whether the injection of autologous MSCs and fibrin through this new engraftment gastroscope can effectively treat EAL.

## Materials and methods

### Animals

Twelve healthy adult male pigs weighing 30–40 kg were sourced from Shanghai Jiagan Biotechnology Co., Ltd. The animals were randomly divided into the MSCs group (n = 6) and the control group (n = 6). The pigs were kept following the Institute’s animal management regulations. The Biomedical Research Ethics Committee of the Second Military Medical University approved the animal experiments (No.IACUC-190162).

### Porcine MSCs isolation and cell culture

A 5-mL bone marrow sample was derived from the ileum of each animal and suspended in 5 mL phosphate-buffered saline (PBS) inside a sterile 15-mL conical tube. The sample was centrifuged at 1500 rpm for 20 min, then the buffy coat was isolated in 5 mL Ficoll (GE Healthcare, USA). The purified cells were harvested and washed twice using aseptic PBS. The samples were centrifuged again, then the supernatant was removed. The cells were resuspended (at the appropriate cell density) in 6-well plates. MSCs were cultured in a mixture of Dulbecco’s modified Eagle’s medium, 100 U/mL penicillin (Gibco, USA), 100 μg/mL streptomycin (Gibco), and 10% fetal bovine serum (Gibco) in a humidified incubator at 37°C and 5% CO_2_. The culture medium was changed every two days. All the experiments were performed using MSCs harvested from passages 3 and 5.

### Flow cytometry analysis

MSCs were blocked with bovine serum albumin for 30 min (Thermo Scientific, USA), then incubated with primary antibodies CD29 (1:400, ab6124; Abcam, UK), CD90 (1:100, ab23894; Abcam), CD34 (1:100, ab81289; Abcam), and CD45 (1:400, ab10558; Abcam) at 4°C overnight. Negative control MSCs were not incubated with the primary antibodies. The samples were washed twice using PBS, then incubated with the corresponding fluorescein 5-isothiocyanate-labeled secondary antibodies in the dark at room temperature for 1 h. Finally, the cells were washed again twice using PBS, centrifuged at 1500 rpm for 5 min, and resuspended in 1.5-mL tubes before flow cytometry analysis.

### Differentiation assays

Specific adipogenesis and osteogenesis media (Gibco) were used to induce MSCs differentiation into adipocytes (2 weeks) and osteocytes (4 weeks), respectively. The differentiation status was verified *via* Oil-red O and alizarin red staining.

### EAL model construction

**Figure 2:**
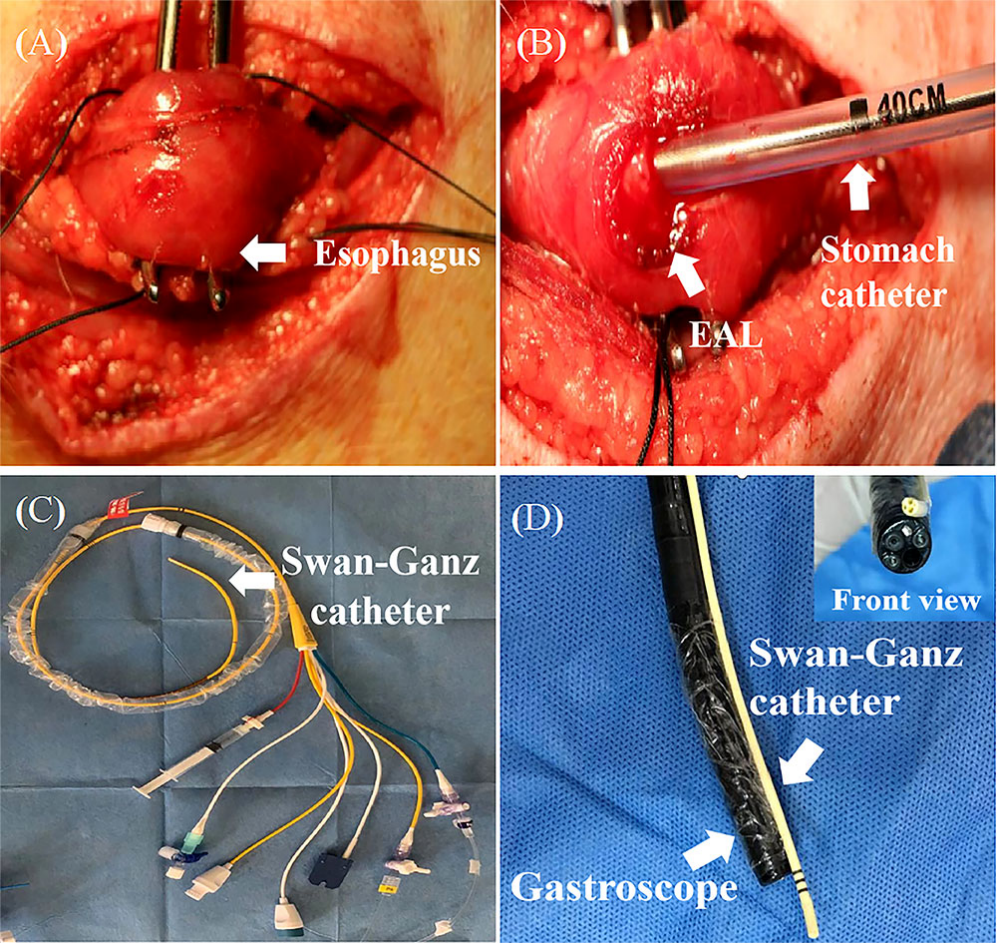
The construction of EAL model. **(A)**, The isolation and exposure of esophagus in pig *via* left neck incision. **(B)**, An artificial leakage and a stomach tube (8 mm caliber) inserted in the neck esophagus. Successful extraction of gastric juice *via* the stomach tube indicated appropriate catheter depth. The catheter was inserted into the digestive tract to an average depth of 35 cm. **(C, D)**, Swan-Ganz catheter fixed at the front end of the gastroscope with its terminal end moved forward by about 1 cm. EAL, esophageal anastomotic leakage.

The animals were tranquilized *via* injection of midazolam (0.25 mg/kg) and intramuscular injection of ketamine (8 mg/kg) to establish the EAL model. The pigs given endotracheal intubation were held in the supine position on the animal operating table for appropriate visualization of subsequent surgery. The pigs were intravenously injected with propofol (4 mg/kg/h) during the procedure to maintain anesthesia. Mechanical ventilation was created to ensure good oxygen saturation and prevent respiratory complications. Left neck incisions were made on all pigs. A leakage of about 9 mm (diameter) was left in the neck section of the esophagus of pigs after isolation and transection. A stomach tube with a caliber of 8 mm was inserted through the leakage to construct the EAL model (**Figures 2A, B**). One end of the catheter was left in the stomach, while the other was fixed to the skin around the neck. Successful extraction of gastric juice *via* the neck side indicated appropriate catheter depth. The catheter was inserted into the digestive tract (average depth; 35 cm) to allow enteral nutrition through the stomach tube. Moreover, broad-spectrum antibiotics were administered intravenously. The stomach catheters were removed one week after the establishment of EAL model, then gastroscopy was performed to assess EAL conditions.

### MSCs engrafting in a fibrin scaffold and delivery to the EAL site by gastroscopy

**Figure 3:**
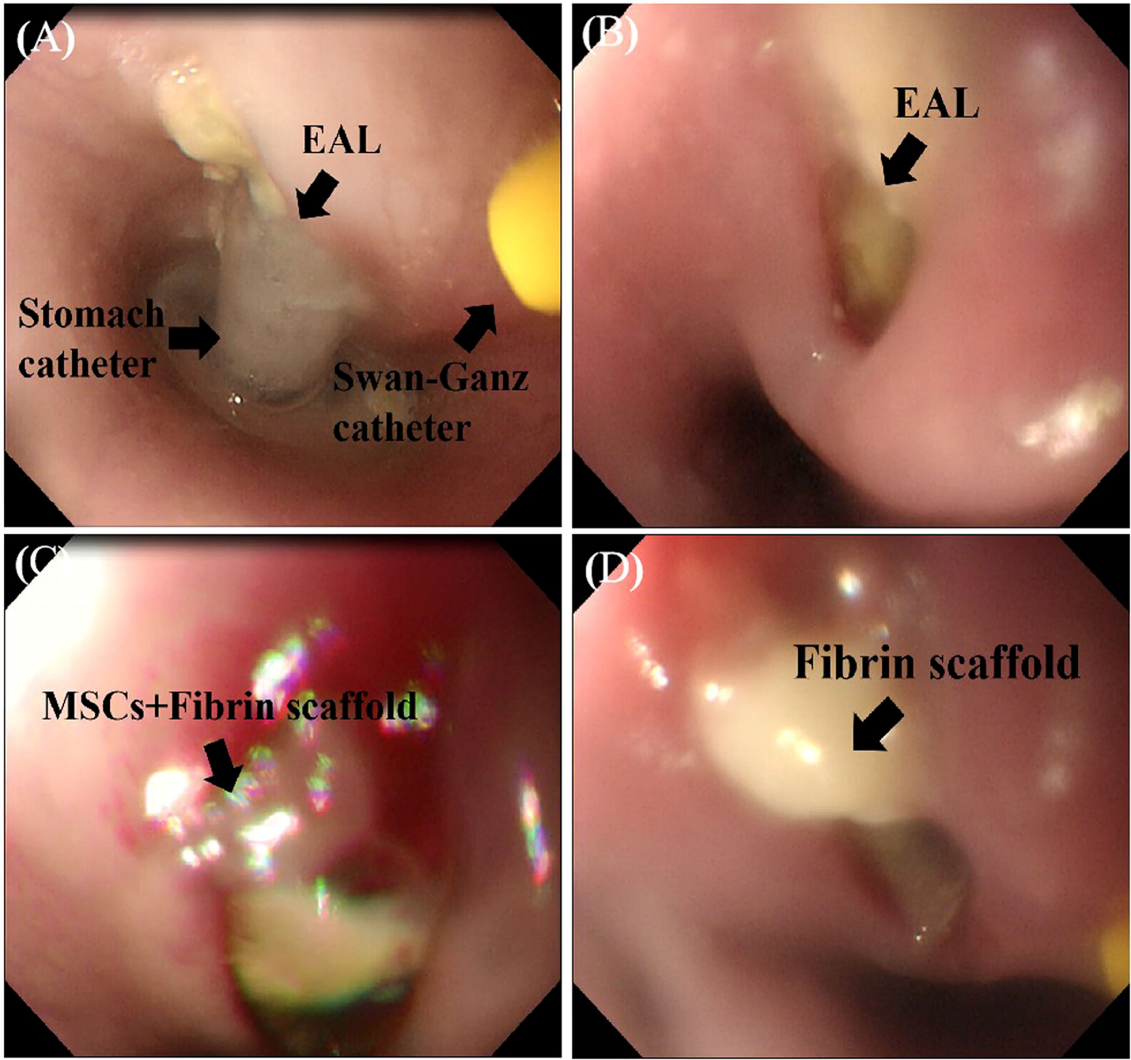
MSCs engrafted in a FS and delivered to the EAL site. **(A)**, The observation of EAL conditions *via* gastroscope after one week. **(B)**, Gastroscope showing EAL after removal of stomach catheter. **(C)**, Injection of 2 mL FS containing 2×10^7^ MSCs (treatment group) around the leakage. **(D)**, Injection of 2 mL FS (control group) around the leakage. EAL, esophageal anastomotic leakage; MSCs, mesenchymal stromal cells; FS, fibrin scaffold.

A Swan-Ganz catheter was fixed at the front end of the gastroscope, with its terminal end moved forward by about 1 cm for good vision and convenient operation (**Figures 2C, D**). The fibrin scaffold was prepared before engraftment by mixing two different solutions. One solution contained lyophilized fibrinogen resuspended in 2 mL dilution buffer, and the other solution contained 40 mM CaCl_2_ mixed with 500 IU/mL thrombin (total volume of 2 mL). The two solutions were mixed at a 1:1 proportion to induce fibrin scaffold synthesis during the engraftment procedure. The pigs were anesthetized, then held in a supine position, and injected with 2 mL fibrin scaffold containing 2 × 10^7^ MSCs (treatment group) or 2 mL fibrin scaffold alone (control group) using gastroscope (**Figure 3**). The animals were turned after the stomach catheters were removed, then held in a prone position for at least 30 min. The animals were fed through a stomach tube for a week, after which they were fed through the mouth.

### Western blotting

Esophagus tissue samples from the graft site were washed with PBS. Cell lysis was used to obtain total proteins on ice for 30 min in SDS buffer containing a protease inhibitor cocktail (1:100). The cell lysates were separated using 10% SDS-PAGE, transferred to polyvinylidene difluoride membranes, then blocked at room temperature for 1 h. The membranes were incubated with primary antibodies against myogenin (1:5000, ab1835; Abcam), alpha-smooth muscle actin (α-SMA, 1:5000, ab7817; Abcam), and β-actin (1:5000, ab8227; Abcam) on a shaker at 4°C overnight. The membranes were incubated with the corresponding secondary antibodies (1:5000, 115-035-003; Jackson Immuno Research, USA) at room temperature for 1 h. Electrochemiluminescnece kit (Thermo Scientific) was then used to visualize the membranes, then exposed to film.

### EAL observation and biological safety evaluation

The animals underwent gastroscopy one month after the injection to evaluate the local EAL status. Healed status was defined as complete occlusion of the mucosal layer that is detected using gastroscopy. Conversely, unhealed status was defined as incomplete occlusion of the mucosal layer with or without purulent exudate. Additionally, liver and kidney function tests were performed using blood from the ear veins in the 2^nd^ week to assess the biological safety of the treatment.

### Histological analyses

The pigs were sacrificed in the 4^th^ week following relevant animal management regulations. The specimens of esophagi at EAL sites were collected, processed, and subjected to histological analyses. The samples were fixed in 10% buffered formaldehyde, dehydrated in an ethanol series, embedded in paraffin, and sliced into 4-μm-thick sections before hematoxylin-eosin (HE) staining. A light microscope was used for histological examinations, and images were taken using a microscopy imaging system.

### Statistical analysis

Data are expressed as the mean ± standard deviation. GraphPad software (GraphPad Inc., USA) was used for all statistical analysis. Student’s *t*-test or paired *t*-test was used to analyze normally distributed variables, while Wilcoxon signed-rank test was used to assess non-normally distributed variables. P < 0.05 was considered significant.

## Results

### MSCs characterization

**Figure 4:**
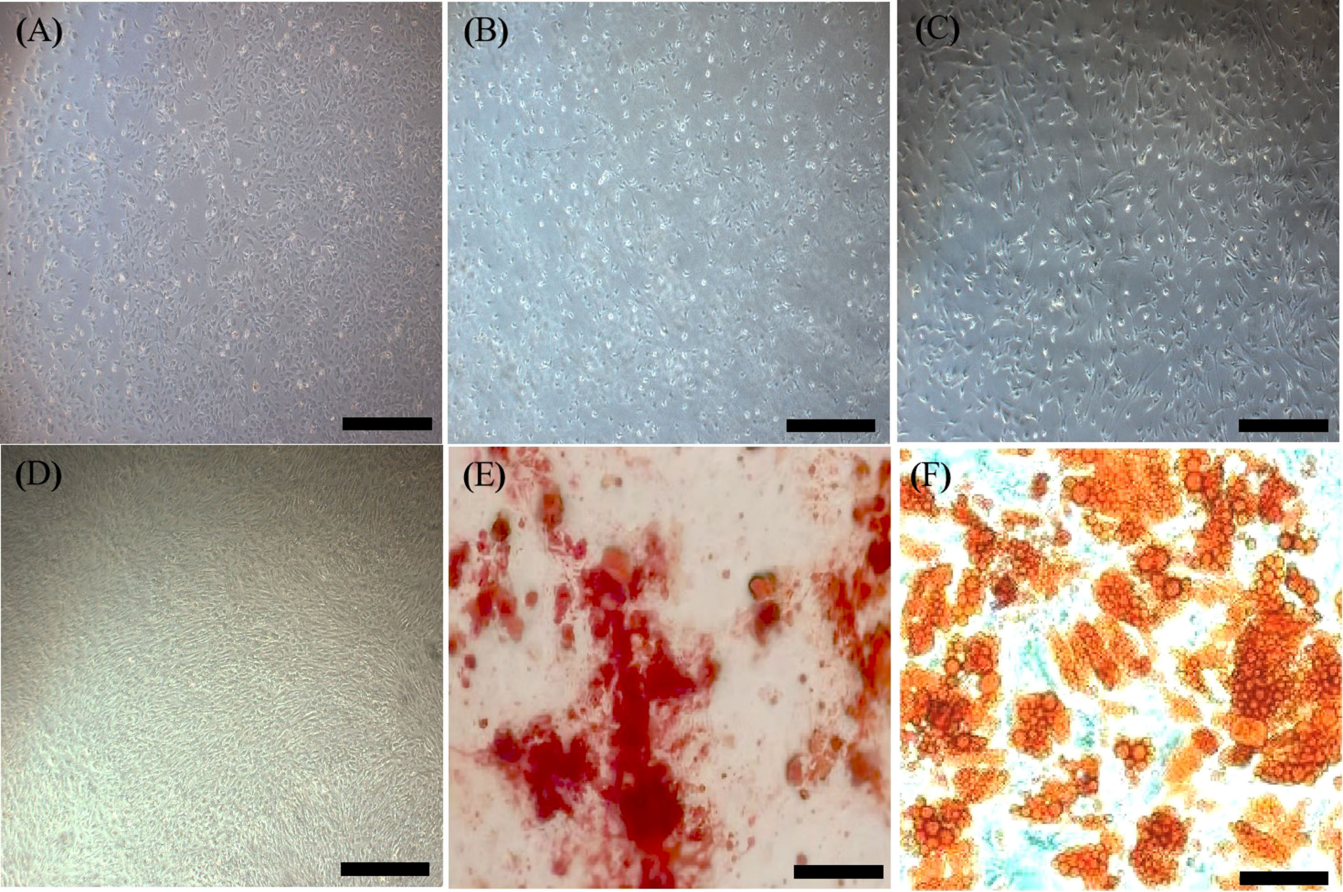
Characterization of MSCs. **(A)**, First generation MSCs (×40, Scale bar=400 µm.). **(B)**, Third generation MSCs (×40, Scale bar=400 µm.). **(C)**, Fifth generation MSCs with a regular shape (×40, Scale bar=400 µm.). **(D)**, Tenth generation MSCs (×40, Scale bar=400 µm.). **(E)**, Alizarin red staining showing osteogenesis (×100, Scale bar=200µm.). **(F)**, Oil red O staining showing adipogenesis (×100, Scale bar=200µm.). MSCs, mesenchymal stromal cells.

An inverted microscope showed that MSCs seeded on plates had a large volume, spherical shape, uneven size, bright cell bodies, and strong refractivity. Some cells started to adhere to the plates after 3 h, and most adhered to the plates after 48 h, showing pleomorphism. The cell growth rate increased after sorting *via* flow cytometry. Adherence began after 2 h of cell seeding and was completed within 24 h. Cell fusion occurred after 3 or 4 days and the time for passage was nearly seven days. The 1^st^ and 3^rd^ generation MSCs were small with irregular shapes (**Figures 4A, B**). The cell morphology was not significantly different after 9 to 10 passages (**Figure 4D**). However, growth gradually slowed after 9 to 10 passages, and cells became large and irregularly shaped, with granular substances becoming increasingly visible. The transplanted cells were harvested at the 5^th^ passage (**Figure 4C**). They had a regular shape, an ordered arrangement, and a spindle-like morphology resembling fibroblasts. Oil red O and alizarin staining showed that MSCs could differentiate into adipocytes and osteocytes, respectively (**Figures 4E, F**).

**Figure 5:**
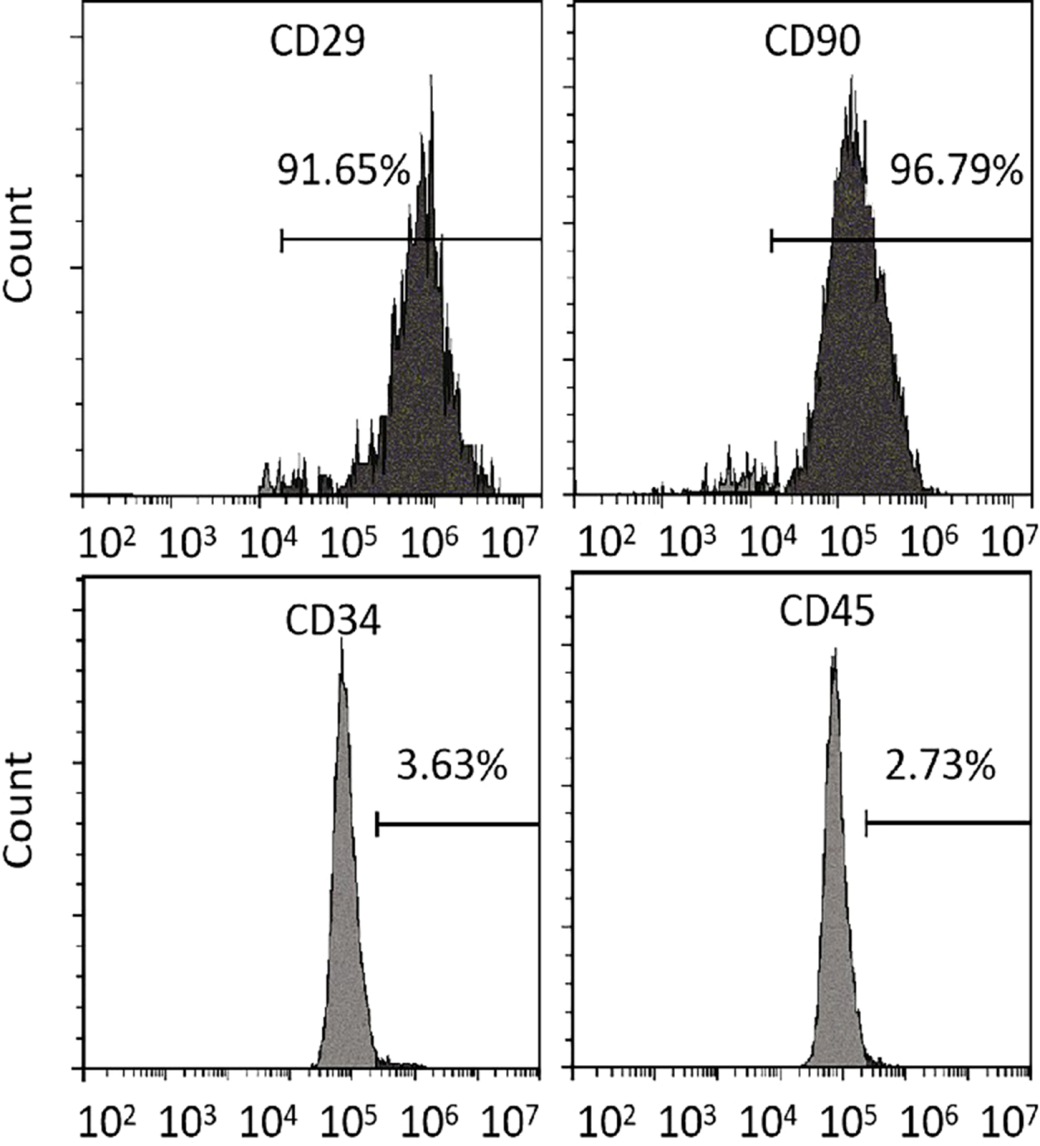
Differentiation assays. Flow cytometry analysis of MSCs-specific (CD29 and CD90) and hematopoietic (CD34 and CD45) cell surface markers. MSCs, mesenchymal stromal cells.

The cell surface markers were analyzed *via* flow cytometry to detect spindle-like 5^th^ passage MSCs. Unlike the negative expression of hematopoietic markers (CD45 and CD34), MSCs-specific cell surface markers (CD29 and CD90) were strongly expressed in MSCs, demonstrating their purity (**Figure 5**).

### Establishment of a porcine model of EAL and biological safety evaluation

The animals underwent gastroscopy one week after establishing the EAL model under anesthesia to observe the local EAL status and evaluate whether the procedure was successful (**Figure 3A**). The nearly circular tissue defect on the esophageal wall after the removal of the stomach catheter showed that the EAL model in pigs was accessible *via* catheter insertion through an artificial leakage for a week (**Figure 3B**). Two pigs in both the MSCs treatment group and control group suffered from localized purulent infections around EALs, indicating that the rate of partial infections near EAL was not statistically different between the two groups (33% vs. 33%, P > 0.05). Routine liver and kidney function tests revealed normal postoperative results in the two groups (**Table 1**). Moreover, there was no significant difference between the two groups, demonstrating the reliability and biological safety of the procedure.

**Table 1:** Biochemical blood profile of Control and MSCs groups of pigs two weeks after treatment.

	Control	MSCs	*P*
Aspartate aminotransferase (IU/L)	22.0 ± 2.6	23.6 ± 3.4	0.74
Alanine aminotransferase (IU/L)	21.8 ± 2.3	26.3 ± 2.8	0.28
Blood urea nitrogen (mg/dL)	9.6 ± 0.8	9.0 ± 0.4	0.48
White blood cells (10^3^/mL)	15.2 ± 1.3	18.4 ± 2.4	0.32
Hemoglobin (g/dL)	9.0 ± 0.3	8.5 ± 0.3	0.31
Platelets (10^3^/mL)	447.2 ± 69.7	375.6 ± 35.8	0.34
Lymphocytes (10^3^/mL)	9.3 ± 0.7	9.4 ± 0.6	0.92
Neutrophils (10^3^/mL)	5.2 ± 0.5	7.8 ± 1.7	0.25
Eosinophils (10^3^/mL)	0.2 ± 0.1	0.4 ± 0.1	0.32

Data are expressed as means ± SEM (n = 6 Control group, n = 6 MSCs group). P values were determined using a two-tailed t-test. AST, aspartate aminotransferase; ALT, alanine aminotransferase; BUN, blood urea nitrogen; HGB, hemoglobin; WBC, white blood cell count.

### Clinical outcomes

**Figure 6:**
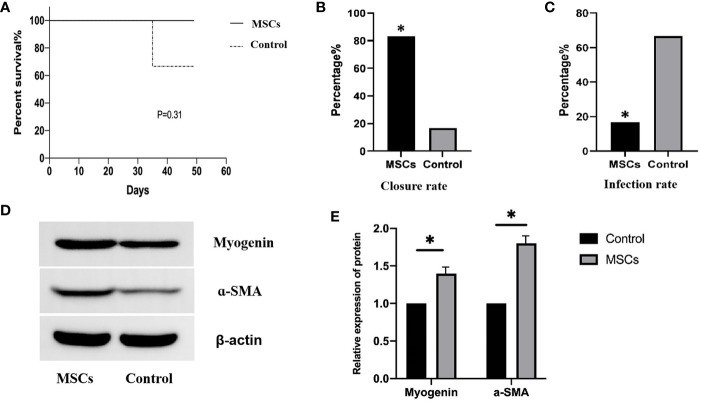
Clinical outcomes for the animals in the two groups. **(A)**, Log-rank test showing mortality rate (P= 0.31) log-rank test. **(B)**, Fisher exact t-test showing the complete closures of EALs in the MSCs group (83.3%) and control group (16.7%). P<0.05. **(C)**, Fisher exact t-test showing the infection proportions of EALs in MSCs group (16.7% (1/6)) and in control groups (66.7%(4/6)) P < 0.05. **(D)**, Western blotting showing myogenin and a-SMA protein expression after MSCs engraftment. **(E)**, Quantitative analysis of the protein expression levels of myogenin and a-SMA. P < 0.05. EAL, esophageal anastomotic leakage; MSCs, mesenchymal stromal cells. *p < 0.05.

**Figure 7:**
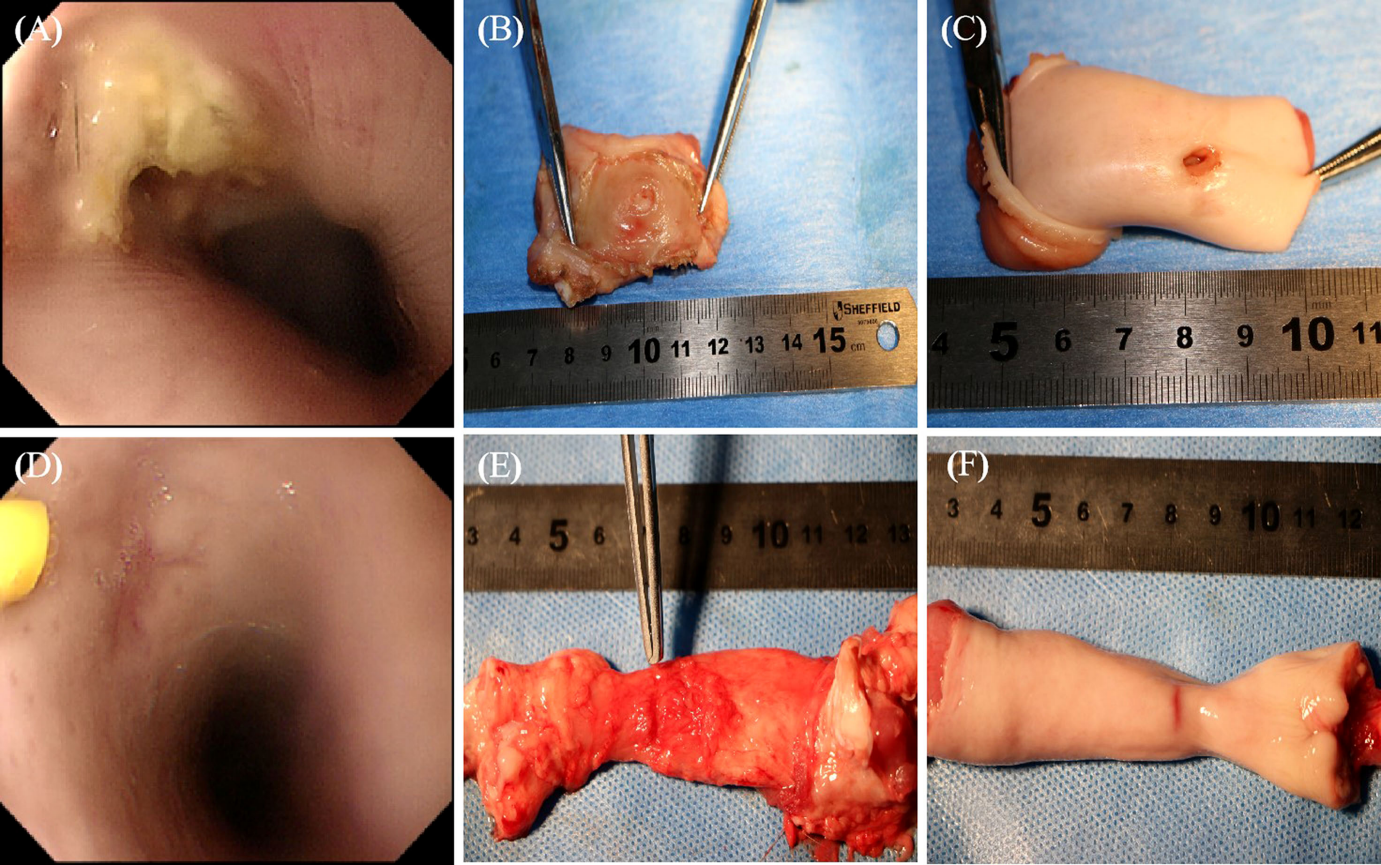
The representative images of specimens of esophagi at EALs with direct vision or gastroscope. **(A)**, The incomplete occlusion of EAL covered with a little purulent exudate observed using gastroscope. **(B)**, The outer layer of leakage (completely occluded) from the outer view. **(C)**, The inner layer of leakage (unhealed status) from inner views, which was mainly involved in the muscular layer. **(A-C)**, Representative images of specimens of esophagi at EALs in the control group. **(D)**, The complete occlusion of EAL covered with some cicatricial tissue detected using gastroscopy. The yellow syringe pointed to cicatricial tissue. **(E, F)**, The completely occluded leakage from both outer and inner views. **(D-F)**, Representative images of specimens of esophagi at EALs in the MSCs group. **(B, E)**, Outer view under direct vision. **(C, F)**, Inner view under direct vision. EAL, esophageal anastomotic leakage; MSCs, mesenchymal stromal cells.

No deaths were recorded in the MSCs group after the 4^th^ week of treatment, while one pig died in the control group, indicating no significant difference in mortality (0.0% vs. 16.7%, P = 0.31; **Figure 6A**). Complete occlusion of the mucosal layer indicated EAL closure. EAL closure occurred in five animals from the MSCs group and only one from the control group (83% vs. 16.7%, P < 0.05; **Figure 6B**). Representative images of esophageal specimens (**Figure 7**) revealed substantial yellow secretion around the EAL in case of infection (**Figure 7A**). EAL infection was detected in one animal from the MSCs group (16.7%) and three from the control group (66.7%)(P < 0.05; **Figure 6C**).

### Detection of protein differentiation markers at the graft site

Esophageal tissues at the graft site were collected to verify the differentiation of engrafted MSCs into muscle cells. The expression of relevant proteins was detected using Western Blotting. The myogenin and α-SMA expressions were significantly higher after MSCs engraftment than in the control group (**Figures 6D, E**), suggesting that MSCs successfully differentiated into muscle cells.

### Histological analysis

**Figure 8:**
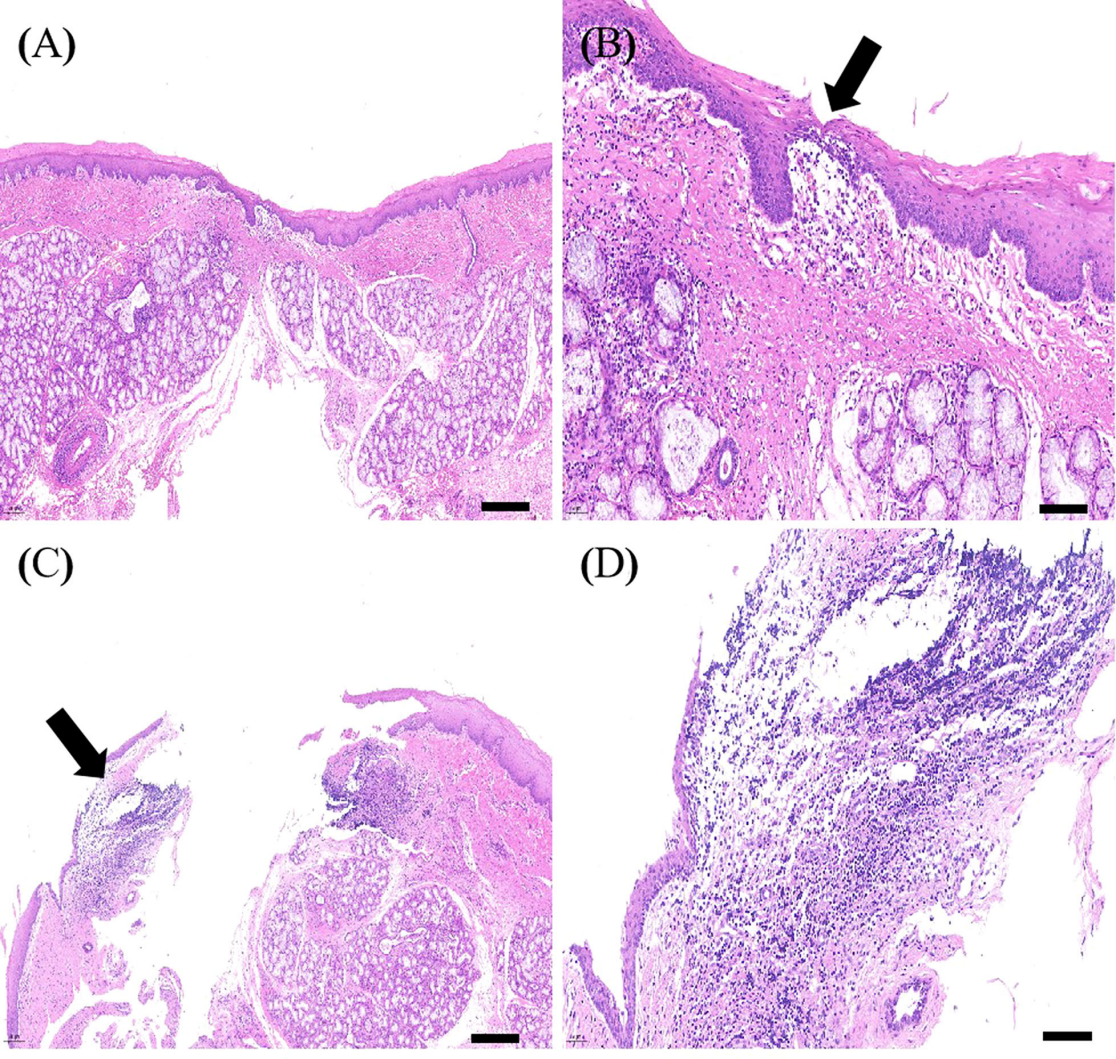
The representative images of HE staining of the esophagi at EALs. **(A)**, The complete occlusion of EAL (Scale bar:200 μm). **(B)**, Inflammatory cells infiltrating around EAL from adventitia to muscular layer (Scale bar:50 μm). **(A, B)**, Representative images of HE staining of the esophagi at EALs in MSCs group. **(C)**, The incomplete occlusion of EAL (Scale bar:200 μm). **(D)**, Dispersive infiltration of inflammatory cells in the full-thickness esophageal tissue (Scale bar:50 μm). **(C, D)**, Representative images of HE staining of the esophagi at EALs in the control group. EAL, esophageal anastomotic leakage; MSCs, mesenchymal stromal cells.

HE staining showed that the esophagi at EALs in the control group had a severe inflammatory response with dispersive infiltration of inflammatory cells. Furthermore, thickening of esophageal tissue, unclear tissue structures, and unhealed leakage were observed in the control group (**Figures 8C, D**). In contrast, infiltration of inflammatory cells was not altered in the MSCs group (**Figures 8A, B**). A light microscope detected tissue structures and complete occlusion of EALs in the MSCs group (**Figure 8**).

## Discussion

EAL is a severe postoperative complication associated with esophagectomy (16, 17). Although various conservative strategies, such as sufficient drainage, use of appropriate antibiotics, and aggressive surgical repair, have been used for EAL treatment, they have achieved unsatisfactory results (18). However, cell therapy for ischemic heart disease, atherosclerosis, and stroke has recently exhibited remarkable success (19).

Although an EAL model has been successfully established using adult rabbits (15), a porcine EAL model, which is more relevant to human studies, has not been established. Herein, a porcine model of EAL was established in pigs based on the rabbit method, whereby a stomach tube is passed through the leakage for a week.

Various adjustments were made to overcome the limitations of the original rabbit procedure. For example, the strong adhesion to tissues and deeper location of EALs in pigs complicates the therapeutic use of MSCs. As a result, the transplantation site was accessed through the internal of esophagus. Furthermore, a new method for engraftment of MSCs *via* gastroscopy was determined for effective EAL treatment. Finally, EALs were successfully established in all animals without significant differences in infection rates between the control and treatment groups.

The gastroscope was combined with a Swan-Ganz catheter to efficiently inject the fibrin scaffold in the digestive tract. This strategy had several advantages. First, the length of this special catheter (110 cm) matched the length of the gastroscope. Second, the soft material could not damage the esophageal tissue. Third, the smaller diameter of the catheter left little residual fibrin scaffold after injection. Therefore, more MSCs could be engrafted and differentiated near the EAL site. Fourth, the above structural features made it possible to fix the catheter at the front end of the gastroscope, with the terminal end located 1 cm in front of the lens, thus ensuring good vision, convenient operation, strong operability, with lower risk of damaging the lens.

Notably, the animal position during surgery promoted EAL healing. The EAL site was located on the ventral esophagus wall when the animals were tied to the operating table at the supine position. However, gravity would have made it difficult to fix and engraft MSCs near the ventral EAL if the fibrin scaffold was injected at the supine position. Therefore, the pigs were turned and held at the prone position for at least 30 min, allowing the fibrin scaffold to deposit around the EAL on the ventral side and letting MSCs adequately permeate the full-thickness of the esophagus wall.

Previous studies have demonstrated that MSCs with differentiation and immunomodulatory abilities participate in tissue regeneration and repair after transplantation (14). However, the potential underlying mechanisms explaining such therapeutic function are unknown. Nonetheless, studies have shown that the treatment effect is achieved by replacing impaired tissues and cells through paracrine synthesis and secretion of several cytokines or inducing cell differentiation (20, 21). Furthermore, exosomes is known to refer to membrane-bound vesicles released from many cells into the extracellular matrix (22). A review article reported (23) that exosomes from MSCs play a key role in regenerative medicine, many of which have been documented repeatedly to have the capacity to recover damaged tissues (24–26).

Although MSCs migrate to surrounding areas after direct transplantation, only a few survive and colonize the target sites (27). Fibrin promotes the retention, proliferation, and differentiation of MSCs. Fibrin also has good biocompatibility, biodegradability, strong operability, and can be easily injected. Furthermore, the three-dimensional structure of fibrin enables tissue reconstruction and repair at later stages of treatment (28, 29). Fibrin combined with vascular endothelial growth factor and hepatocyte growth factor promotes the secretion of immunoregulatory factors by MSCs, thus attenuating the inflammatory response (30). Therefore, clano-transptation of MSCs and fibrin is crucial for a better outcome.

In this study, western blotting revealed that myogenin and α-SMA expressions were significantly higher in the MSCs groups than in the control group, indicating that MSCs differentiates into myoblasts. Histological HE staining showed that the infection level in EALs was significantly lower in the experimental group than in control, possibly due to the immunoregulatory function of MSCs after implantation (31).

Furthermore, the closure rate of EAL was significantly higher in the MSCs group than in the control group (83.3% vs. 16.7%). In contrast, the infection rate was significantly higher in the control group than in the MSCs group (16.7% vs. 66.7%). These findings suggest that the proposed method allows the safe and reliable occlusion of EAL, thus preventing the onset of local infections. The successful outcome of the proposed EAL treatment could be because of the fibrin-dependent inhibition of MSCs migration, immunomodulatory effect of MSCs, influence of MSCs on the reconstruction of the extracellular matrix, and the absence of any impairment caused by reoperation.

Compared with mortality, the survival rate was not statistically different between the treatment and control groups (0.0% vs. 16.7%). However, the higher infection rate and lower closure rate of EAL in the control group indicate that MSCs therapy has a protective effect. Nevertheless, a study with a larger sample size and longer experimental period is needed to assess the survival rate between the two groups. Meanwhile, the routine blood parameters, liver and kidney function tests were not significantly different between the treatment and control groups, indicating the biological safety and reliability of the MSCs treatment.

Taken together, these results demonstrate that the combined implantation of MSCs and fibrin scaffold *via* the proposed method facilitates the repair and occlusion of EAL. Therefore, the proposed method could be best for EAL treatment. Moreover, the method can significantly reduce infection rates, making it unnecessary to perform another surgery. This would reduce the mortality rate since it reduces the risks associated with anesthesia and reoperation. Meanwhile, a single intervention can reduce hospitalization time and lower the corresponding costs.

## Conclusion

In summary, a porcine model of EAL was successfully developed by accessing the transplantation site through the esophagus. The implantation of MSCs in FS *via* the novel engraftment gastroscope can also promote the repair and occlusion of EAL, suggesting that it could be a promising therapeutic strategy for EAL treatment.

## Data availability statement

The original contributions presented in the study are included in the article/supplementary material. Further inquiries can be directed to the corresponding author.

## Ethics statement

The animal study was reviewed and approved by the Biomedical Research Ethics Committee of the Naval Medical University.

## Author contributions

HZ designed the study. YH and HC wrote the manuscript. YH, HC, XX, YY, WC, and XL conducted data collection and analysis. HZ, YH, and HC revised the manuscript and finally approved the submitted and published versions. HZ is the guarantor of this work and has full access to the data, and takes responsibility for the integrity of the data and the accuracy of the data analysis. All authors contributed to the article and approved the submitted version.
